# Assessment of serum L-fucose in brain tumor cases

**DOI:** 10.4103/0972-2327.61274

**Published:** 2010

**Authors:** S. Manjula, Flama Monteiro, Annaya Rao Aroor, Suryanarayan Rao, Raja Annaswamy, Anjali Rao

**Affiliations:** Department of Biochemistry, Yenepoya Medical College, Mangalore, India; 1 Department of Biochemistry, Mandya Institute of Medical Sciences, Mandya, Karnataka, India; 2 Department of Medical Pharmacology & Physiology, University of Missouri School of Medicine, Columbia MO 65212, India; 3 Department of Neurology, Kasturba Medical College, Manipal, Karnataka, India; 4 4Biochemistry, Kasturba Medical College, Manipal, Karnataka, India

**Keywords:** Brain tumors, cysteine hydrochloride, glycosylation, L-Fucose

## Abstract

**Background:**

Glycosylation of altered tumor cell in relation to cellular heterogeneity in human intracranial tumors remains relatively unexposed. Serum protein-bound carbohydrate, L-Fucose is reported to be overexpressed during tumor progression by many investigators. Therefore, there is a need to determine the diagnostic, prognostic, functional significance of glycoprotein elevations in various cases of tumors.

**Objective:**

The objective of the present study was to evaluate the clinical utility of serum L-fucose in patients with brain tumor.

**Materials and Methods:**

Serum glyco-conjugate levels were estimated in 99 patients with brain tumors. Estimation of L-fucose was carried out colorimetrically by the method of Winzler using cysteine hydrochloride.

**Results:**

There was a significant increase in L-fucose level in most of the patients. In the posttreatment cases, the L-fucose levels were apparently low compared to preoperative values.

**Conclusion:**

Our results showed that the rise in serum L-fucose may be used as a general marker for brain tumors in addition to other markers.

## Introduction

Cell surface glyco-conjugates are important in relation to cancer, because many of the altered properties of cancer cells are expressed at the cell surface. Warren *et al*.[1] had observed that only traces of tumor characteristic sialofucosyl glycopeptides are found in the serum of healthy subjects, while it is found in high concentrations in malignant transformed cells. Growth factor receptors and glyco-conjugate molecules are able to interact with each other and this interaction usually results in modulation of growth factor receptor—mediated signaling and the biological function of the cell.[2] Glyco-conjugate molecules expressed at the plasma membrane of mammalian cells have also been reported to be associated with cell-to-cell adhesion, tumor progression, and metastasis.[3] Measurements of protein-bound carbohydrates have been used as an index to glycoprotein levels that may be valuable in establishing diagnosis, staging of disease, detecting metastases, identifying patients at high risk for recurrence and evaluating therapeutic response. The carbohydrate of glycoprotein is composed of a relatively small number of different monosaccharides one of them is L-fucose, which is usually a terminal sugar. It is one of the essential sugars that body requires for optimum function of cell-to-cell communication.[4,5] Normally, it is present with low concentrations in normal serum, but is increased in cancer and other diseases.

Elevated levels of serum protein-bound fucose have been reported in patients with malignant disease and in certain benign disorders.[6,7] Markedly, elevated fucose levels have been assessed in cancer of the breast,[8–11] gynecological cancers, leukemia,[12 ] small cell lung cancer, and ovarian cancer.[6,13–15] Abnormal metabolism of L-fucose in hepatocellular carcinoma was observed.[16] An increase in fucose levels was also observed in cardiovascular disorders[ 17] and in patients with depression.[18] A metastasis model seems to show a higher uptake of fucose tracers compared to a primary model.[19] L-Fucose in type 2 chain H antigen in oral premalignant lesions may prove helpful in early diagnosis of epithelial cancer.[20] Radioactive L-fucose labeling of brain tumors in rats was used to identify 9L brain tumors.[21] The use of glyco-conjugates or their derivatives may represent a new approach to modulate the proliferative behavior of tumors that overexpress growth factor receptors such as brain tumors. However, there are no reports available on the L-fucose glycoconjugate levels in patients with intracranial tumors. Therefore, in the present study, L-fucose estimation was carried out in the serum of brain tumor patients.

## Materials and Methods

### Patients with brain tumors

Ninety nine patients with brain tumors, between 10–75 years of age, formed the case material for this study. All were histopathologically confirmed for their neurological status. There were 61 male and 38 female patients. A total of 17 follow-up cases were studied, when the patients revisited the hospital for further advice and guidance from the neurosurgeon. In this study, various types of brain tumor included were: glioma, meningioma, acoustic neurinoma; and other types which included a few cases of medulloblastoma, secondary tumors, and craniopharyngioma. The healthy volunteers( 35 of them), age and sex matched, were taken as control subjects for the present study.

### Sample collection

Blood was collected by venipuncture from the patients prior to surgery and from age and sex matched healthy volunteers. Further serum samples were obtained from these patients when they reported for follow-up between 6-20 weeks at their convenience. The samples were collected on different days because of patients visiting hospital on different days. The serum was separated, centrifuged and stored at –70°C.

### Determination of L-fucose in serum

L-Fucose was estimated according to the method of Winzler[22] using cysteine hydrochloride. L-Fucose was assayed by dissolving ethanol-precipitated proteins of serum in alkali, heating with sulphuric acid and determining the color after addition of cysteine. Standard L-Fucose was procured from Sigma Chemical Company, MO, US. The color produced by hexoses under these conditions was corrected by determining absorbance at 400 nm and 430 nm.

Statistical analysis was carried out according to Student's paired and unpaired *t*-tests. The results were considered significant if the *P* value was less than 0.05.

## Results

The mean values obtained for serum protein bound fucose in healthy control subjects, benign and malignant tumors of the brain are shown in Table 1. The mean value for protein bound fucose in patients having meningioma was significantly higher (*P <* 0.02) compared to healthy controls. The mean L-fucose in glioma patients was also high (*P <* 0.05) compared to the control values, while there was no significant change in the mean values in acoustic neurinoma and other types.

**Table 1:** Serum L-Fucose levels of intracranial tumor patients (Mean ± SD)

Clinical condition	Number of cases	L-Fucose mg/dl
Control	35	16.87 ± 6.5
Glioma	47	21.46 ± 11.05^b^
Meningioma	22	23.03 ± 12.07^a^
Acoustic neurinoma	14	20.40 ± 10.61
Other types	16	21.38 ± 12.16

a *P* < 0.02;

b *P* < 0.05. Significantly different from control

There was a marked increase in the serum L-fucose levels when a comparison of the control group versus benign tumors (*P <* 0.05) and malignant tumors (*P <* 0.05) was made. But no significant change was observed in benign versus malignant brain tumors [Table 2].

**Table 2:** Comparison of L-fucose levels between benign and malignant intracranial tumor cases

Clinical condition	Number of cases	L-Fucose mg/dl
Control	35	16.87 ± 6.5
Benign	47	21.85 ± 11.93
Malignant	52	21.47 ± 10.85

NS = Non significant, Control vs Benign: *P* < 0.05; Control vs Malignant: *P* < 0.05, Benign vs Malignant: NS

In Table 3, parameters of serum L-fucose levels in preoperative and posttreatment cases of glioma patients are presented. The treated cases during the follow-up, showed significant decrease in the mean values of fucose. However, when individual cases were compared, both decrease and increase in the levels of protein bound fucose in serum, was seen. In patients showing no recurrence after surgery and treatment, the L-fucose levels remained high in medulloblastoma and meningioma compared to preoperative values [Table 3]. In a single case of acoustic neurinoma, a mild decrease in protein bound fucose level was found [Table 3].

**Table 3:** L-Fucose levels in the serum of preoperative and post-treatment cases of brain tumor patients

Diagnosis	Sample collected	L-Fucose(mg/dl)		
		
Serial No. and Sex	after surgery (in days)	Preoperative	Post-treatment	Mean± SD
Glioma				
1. M	30	18.0	16.0	Preoperative 23.42 ± 8.9 (n = 16)
2. M	30	21.33	24.0	
3. M	30	18.66	28.0	
4. M	30	28.0	21.33	
5. M	30	42.66	29.33	
6. M	30	24.23	19.0	
7. F	30	29.33	21.33	
8. M	60	24.0	24.0	
9. F	60	22.66	32.0	Post-treatment 22.21 ± 5.69^c^ (n = 16)
10. M	90	13.33	27.2	
11. M	90	28.0	16.0	
12. F	90	13.33	25.33	
13. F	90	26.66	25.33	
14. M	150	8.0	14.66	
15. F	150	37.9	20.0	
16. M	180	18.66	12.0	
Meningioma				
17. F	30	-	37.33	Preoperative 24.84 ± 15.48 (n = 4)
18. M	60	42.66	20.0	
19. M	120	22.66	22.66	Post- treatment 25.33 ± 7.6^b^ (n = 5)
20. M	120	5.33	28.0	
21. F	250	28.8	18.66	
Acoustic neurinoma				
22. M	120	18.66	16.0	-
Medulloblastoma				
23. M	60	24.0	30.66	Preoperative 20.22 ± 4.9 (n = 3)
24. M	60	22.0	13.33	Post- treatment 28.66 ± 14.74^a^ (n = 3)
25. F	210	14.66	46.66	

M = Male, F = Female, N = Number of samples

a *P* < 0.01

b *P* < 0.02

c *P* < 0.05. Significantly different from control, No significant change with the preoperative values

## Discussion

In the present study, serum fucose levels were found to be elevated in most of the intracranial tumor patients. Winzler[22] suggested that in view of multiplicity and heterogeneity of serum glycoprotein, it is likely that different components may have different sites of origin and reflect different pathological process. L-Fucose is found in many glycolipids and glycoproteins including several families of blood group antigens.[23] Changes have been detected in the fucosylation pattern of these molecules in the tissue of cancer patients, due to fucosyl transferase activity, which is especially high in the serum of patients suffering from high malignant or metastatic tumors, such as colon carcinoma, breast, and liver cancer.[24,25] Serum levels of free L-fucose of cancer patients had significantly higher levels than healthy persons.[25] The elevated levels of fucose have been attributed to tissue destruction and tissue proliferation or may arise from liver, reflecting a process of protein synthesis. However, various views have been put forward by several workers in support of increase in serum glycoproteins in malignancy and other diseases.[6–19,26–32] The increase in levels may be due to local synthesis and liberation of the glycoproteins by tumor cells into the blood or it is a manifestation of the generalized effects of the tumor on the body metabolism.

Serum fucose level is considered a better biochemical tumor marker than sialic acid level in oral squamous cell carcinoma.[28,29] Some studies have concluded that fucose and mannose appeared to be the most effective of the essential sugars when it came to slowing the growth of cancer cells.[4,5] Although, mean serum fucose levels after therapy was found to be decreased, some of the follow-up cases showed an elevation after radiation and chemotherapy in the present study. A similar observation was also made by Dutta *et al.,* in breast malignancy.[33] A rise in postoperative serum fucose level has been observed due to the development of distant metastases, or due to surgical trauma, severe inflammation and tissue necrosis in breast malignancy.[10] Apart from fucose being a prospective tumor marker, it is found to be a powerful immune modulator. It is distributed in macrophages, which are critically important to immune function. There have been numerous well-documented benefits for its necessity in immune function, especially that of an overactive immune system, the cause of autoimmune disorders. Fucose is showing promise in its ability to normalize immune function.[4,5] Therefore, altered levels of serum protein bound fucose may be indicative of both tumor burden and inflammatory response. In this regard, recent studies on inflammatory cytokine levels in brain tumors showed persistence of increased cytokine levels during follow up, thus favoring mounting of inflammatory response during postoperative period.[34] However, the rise in L-fucose in brain tumors suggests that it may be used as a general marker in addition to other markers for brain tumors but it may not be useful for the monitoring of prognosis in brain tumors.
